# Hyperuricemia as an Early Indicator of Cardiovascular Risk in the General Population

**DOI:** 10.3390/jcm14227922

**Published:** 2025-11-08

**Authors:** Fady Al-Azem, Bastian Schrader, Joachim Schrader, Albrecht Elsässer, Bernhard Vaske, Stephan Lüders

**Affiliations:** 1 Department of Internal Medicine and Nephrology, St.-Josefs-Hospital, 49661 Cloppenburg, Germany; prof.dr.schrader@t-online.de (J.S.); vaskebernhard@t-online.de (B.V.); stephan.lueders@kh-clp.de (S.L.); 2 Department of Cardiology, University of Oldenburg, 26133 Oldenburg, Germany; bastian.schrader@uni-oldenburg.de (B.S.); albrecht.elsaesser@uol.de (A.E.); 3 Department of Nephrology and Rheumatology, University Medicine Göttingen, 37075 Göttingen, Germany

**Keywords:** hyperuricemia, coronary artery disease, hypertension, atrial fibrillation, chronic kidney disease, myocardial infarction

## Abstract

**Background**: This prospective cohort study examines the association between hyperuricemia (HU) and cardiovascular diseases. The aim of the current study was to assess whether there might be a correlation between hyperuricemia and cardiovascular risk and future cardiovascular events. **Methods**: We analyzed data from 4082 participants, dividing them into two groups based on serum uric acid levels. **Results**: Our findings reveal that participants with elevated serum uric acid or xanthine oxidase inhibitor (XOI) therapy had a significantly higher incidence of cardiovascular events such as coronary artery disease (8.4% vs. 3.3%), stroke (2.6% vs. 1.2%), heart failure (3.4% vs. 0.9%), and chronic kidney insufficiency (4.5% vs. 1.9%) compared to those with normal uric acid levels. Moreover, group 2, which had higher serum uric acid levels, also exhibited a higher burden of established cardiovascular risk factors, including hypertension, obesity, and diabetes. These results support the hypothesis that HU is not only a marker for metabolic dysfunction but may also serve as an independent risk factor for cardiovascular morbidity and mortality. **Conclusions**: We propose that routine measurement of uric acid levels could be a valuable tool for early identification of high-risk cardiovascular patients, particularly in individuals with multiple metabolic risk factors. Further prospective studies are needed to explore the potential benefits of early XOI therapy in reducing cardiovascular events.

## 1. Introduction

Cardiovascular diseases are among the leading causes of morbidity and mortality worldwide, playing a central role in both preventive and curative medicine [1,2]. Established risk factors such as arterial hypertension, diabetes mellitus, smoking, and dyslipidemia have been extensively studied and play a key role in patient risk stratification [3,4]. Over the years, HU has gained increasing recognition as a potential risk factor for cardiovascular diseases and continues to receive scientific attention. It was first described by Garrod in the 19th Century in relation to gout [5]. Since the mid-20th century, evidence has accumulated linking elevated uric acid to hypertension, obesity, and atherosclerosis [6]. In recent decades, the prevalence of HU has steadily increased, presumably related to changes in dietary habits, as well as the rising numbers of overweight individuals and cases of metabolic syndrome [7,8]. Up until the 19th century, the treatment of HU and gout consisted mainly of weight reduction and a low-purine diet. Today, the central component of treatment involves pharmacotherapy with XOIs [9]. The etiology of primary HU is multifactorial and involves overproduction, increased intake, or reduced renal excretion [10,11]. However, there are also many different underlying pathologies leading to secondary HU resulting in higher production of uric acid, mainly because of an increased number of damaged or destroyed cells. Reasons mainly include drugs (e.g., immunosuppressive agents, tuberculostatic drugs, diuretics) or proliferative diseases inducing a high turn-over of cells (leukemia, hyperthyreosis).

HU promotes systemic inflammation, which is particularly associated with vascular dysfunctions [12]. It is described in the current literature in connection with metabolic syndrome, obesity, and type II diabetes mellitus and may act as an exacerbator of metabolic dysfunctions [13]. Elevated serum uric acid correlates with increased xanthine oxidase activity, which impairs vasodilation and thus contributes to the development of arterial hypertension [14,15]. Moreover, it promotes the formation of free oxygen radicals, which enhance inflammation in the vascular walls [8]. This results in a higher incidence of macroangiopathies such as coronary artery disease (CAD) and Peripheral artery disease (PAD) [16]. Additionally, it contributes to the development of heart failure and cardiac arrhythmias, such as atrial fibrillation [17,18,19,20].

Since the 1980s, HU > 10 mg/dL, regardless of the presence of gout, has been considered an absolute indication for treatment, based on the assumption that very high serum uric acid levels contribute to the development of cardiovascular events [21]. The effectiveness of treating asymptomatic HU to reduce cardiovascular events remains controversial. Different guidelines vary their recommendations regarding HU. The German Society of Rheumatology suggest treatment when organ manifestations such as arthropathy or nephropathy are present, or in cases of asymptomatic HU with a serum level > 10 mg/dL [9]. The American College of Rheumatology recommends the use of XOIs in close conjunction with cardiovascular comorbidities, being less conservative regarding treatment of asymptomatic HU [22,23]. Since 2011, the Japanese Society for Gout has recommended early treatment, considering HU as a cardiovascular risk factor, even in the asymptomatic stage. Both the KDIGO and the European Society of Cardiology acknowledge HU as a cardiovascular risk factor, but recommend treatment only after confirmed organ manifestation [24]. Recent reviews emphasize the complex and conflicting evidence regarding cardiovascular implications of HU and the safety of urate-lowering therapy [25]. Although HU is associated with the development of established risk factors such as arterial hypertension, the current evidence is insufficient to recommend early treatment [26]. However, a recent meta-analysis by Zheng et al. recently found HU to be associated with new-onset hypertension, total CVD, stroke, CHD and CKD [27]. Mechanistically, uric acid is implicated in promoting inflammation, oxidative stress, endothelial dysfunction, and activation of renin-angiotensin system, contributing to cardiovascular pathology [28]. Therefore, to shed further light onto the debated question of whether HU can be considered an independent risk factor for cardiovascular diseases or whether it merely coexists with other more established risk factors such as diabetes mellitus, obesity, and hypertension, we conducted an analysis of patients from the prospective ELITE study [29,30]. The study aimed to assess the potential role of uric acid as a stand-alone risk factor for cardiovascular events such as coronary artery disease, stroke, atrial fibrillation, peripheral arterial disease, and heart failure. Using a large cohort from the Oldenburg Muensterland region, we examined how HU correlates with established risk factors such as hypertension, diabetes, lipid disorders, obesity, and smoking. Additionally, the impact of factors such as gender, age, and comorbidities on these associations was investigated. Furthermore, through a detailed analysis of the dietary habits of the 4000 participants in the study, the relationship between diet and HU was explored in greater detail.

## 2. Materials and Methods

### 2.1. Study Design and Population

The data presented are from the ELITE study (DRKS-No.: 00 006 813), a prospective cohort study conducted over 4.4 years. All participants with available baseline serum uric acid measurements, complete baseline data, and follow-up information were included regardless of their underlying comorbidities even if gout was present at baseline. Individuals who did not attend follow-up were excluded. A total of 4082 patients with normal or elevated serum uric acid levels were divided into two groups. Clinical endpoints such as mortality, myocardial infarction, stroke, heart failure, and other cardiovascular events were recorded annually.

### 2.2. Recruitment

Participants were recruited through newspaper advertisements, invitations to employees of public institutions, e.g., the University of Oldenburg, and invitations sent to randomly chosen citizens of the district of Cloppenburg aged 50 years or older obtained from the residents’ registration office. Patients received no financial compensation, informed consent for the participation was obtained prior to the inclusion, patients could draw back their informed consent at any point of the study. Any patient willing to participate aged 18 years or older was included. Exclusion criterion was draw-back of consent or losing to follow-up. All data was obtained and analyzed anonymously. Financial support was obtained by regional companies, health insurances and public institutions.

### 2.3. Parameters Controlled

Data were collected on age, sex, health status, pre-existing conditions (e.g., hypertension, diabetes, CAD), medication use, and lifestyle (diet, physical activity). Cognitive functions were assessed using the DemTect test.

### 2.4. Cardiovascular Risk Factors

- BMI ≥ 30 kg/m^2^
- LDL > 130 mg/dL
- Smoking
- Diabetes Mellitus: HbA1c > 6.5% or pharmacological treatment
- Blood pressure ≥ 140/90 mmHg

### 2.5. Physical Activity

- Regular physical activity: ≥ 1× daily or 2–3× per week
- Moderate activity: 1×/week or every 2 weeks
- Rare activity/Inactivity: 1×/month or less

### 2.6. Cognitive Impairment (DemTect)

- Physiological: >13 points
- Pathological: 0–12 points

### 2.7. Laboratory Parameters

Creatinine, eGFR, uric acid, glucose, HbA1c, LDL, HDL, lipoprotein (a), iron, and liver values were recorded. HU was considered diagnosed when the serum uric acid level exceeded the threshold of >7 mg/dL for men and >5.7 mg/dL for women. Although current guidelines recommend a single threshold irrespective of sex, large epidemiological studies report higher baseline levels in men, supporting the use of sex-specific cut-offs [29,30].

### 2.8. Follow-Up

Annual follow-up visits were conducted, and computer-generated individual reports on cardiovascular risk profiles were obtained.

### 2.9. Statistical Analysis

The data were analyzed using SPSS (Software version IBM SPSS 29.0). Chi-square tests were used for categorical variables, and the *t*-test for metric variables. To account for multiple comparisons in the univariate analyses, Bonferroni correction was applied. Multivariate logistic regressions were employed to evaluate the influence of multiple factors on cardiovascular events. A *p*-Value < 0.05 was considered statistically significant.

## 3. Results

Table 1 presents the baseline characteristics of the two groups. Of the 4082 participants, 805 individuals (19.7%) had elevated uric acid levels or were undergoing pharmacological treatment for known HU (Group 2), while 3277 (80.3%) had normal levels (Group 1; *p* < 0.001). In Group 2, approximately 15% received XOI therapy. Participants in Group 2 were more likely to be male (61% vs. 48.5%) and, on average, older (58 vs. 52 years; *p* < 0.001).

In Group 1, 1.64% of patients reported a stroke, 2.9% had CAD, 1.15% had a myocardial infarction, 1.7% had heart failure, 2.8% had atrial fibrillation, 4.9% had type 1 or type 2 diabetes, 56.4% had arterial hypertension, 4.7% had PAD and 1.92% had chronic kidney disease (CKD). In Group 2, the values for these pre-existing conditions were consistently higher: 3.4% for stroke, 9.3% for CAD, 4.9% for myocardial infarction, 4.4% for heart failure, 6.8% for atrial fibrillation, 12.8% for diabetes mellitus, 82.7% for arterial hypertension, 7.3% for PAD, and 14.1% for CKD, with differences being statistically significant in all cases.

The second section of Table 1 presents the risk profiles of Group 1 and Group 2. In summary, significant differences were observed between the two groups in several relevant clinical parameters, particularly in terms of BMI, arterial hypertension, HbA1c, diabetes status, and lipid profiles, with Group 2 showing a more burdened cardiovascular risk profile. Only smoking showed no significant difference between the groups.

**Figure 1:**
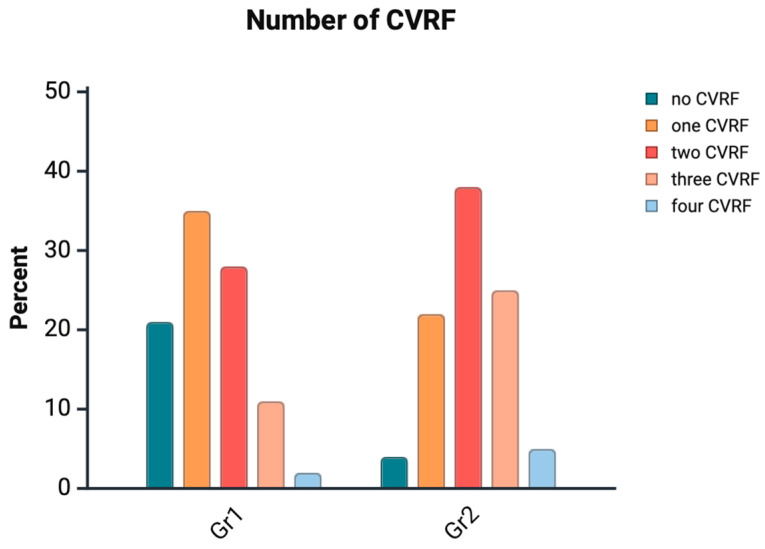
Number of Cardiovascular Risk Factors (CVRF).

A significant difference was observed in the number of cardiovascular risk factors per participant between Group 1 and Group 2. In Group 1, 20.7% of participants had no risk factors, while 34.9% had one, 27.6% had two, 10.8% had three, and 1.5% had four risk factors. In contrast, in Group 2, only 4.3% had no risk factors, 21.5% had one, 38% had two, 25% had three, and 5.2% had four risk factors (Figure 1).

In Group 1, 43.1% of participants reported engaging in regular vigorous physical activity. A moderate level of physical activity was reported by 28.6%, while 28.3% engaged in little to no physical activity (*p* < 0.001). In Group 2, 33.5% reported engaging in regular vigorous physical activity, 29.8% participated in moderate physical activity, and 36.7% had little to no physical activity in their daily routine (*p* < 0.001) (Table 1).

A low level of fruit intake was noted in 15.3% of individuals in Group 1 and in 17.3% of those in Group 2. The proportion of participants with moderate fruit intake was nearly identical between groups (24.7% in Group 1 vs. 25% in Group 2). The majority in both groups reported a high consumption of fruit, accounting for 58.7% in Group 1 and 56.1% in Group 2. Low meat intake was reported by 20.9% of Group 1 and 16% of Group 2. Most participants consumed a moderate amount of meat, with 65% in Group 1 and 61.3% in Group 2. A higher intake of meat was slightly more prevalent in Group 2 (22.7%) than in Group 1 (13.3%) (Table 1).

At the beginning of the study, Group 1 scored an average of 16.3 points (SD 2.2) on the DemTect, which increased to 16.6 points (SD 2.2) by the end of the study. Group 2 started with 15.5 points (SD 2.8) and reached 16.4 points (SD 2.2) at the study’s conclusion (Table 1).

During the study period, the overall mortality rate was 2% in Group 1 and 8% in Group 2 (*p* < 0.001). Cardiovascular events occurred in 11% of participants in Group 1 and 22% in Group 2 (*p* < 0.001). Participants with HU had a significantly higher incidence for cardiovascular endpoints. The excess risk was most pronounced for CAD (8.4% vs. 3.3%), stroke (2.6% vs. 1.2%), CKD (5.5% vs. 1.9%) and myocardial infarction (3.1% vs. 1.3%). Please see Table 2.

In a multivariate analysis, the impact of age, diabetes mellitus, and HU on the clinical endpoints was evaluated. Age emerged as a significant predictor in all models (*p* < 0.001). Diabetes showed significant associations with CAD, stroke, CKD, myocardial infarction and heart failure. HU was significantly associated with the same endpoints except heart failure (Table 3).

## 4. Discussion

The results of this prospective cohort study underline that there is a significantly higher prevalence and incidence of cardiovascular diseases and associated risk factors in individuals with elevated serum uric acid levels or those already undergoing XOI therapy. In our study, participants with HU experienced significantly higher rates of CAD (8.4% vs. 3.3%), stroke (2.6% vs. 1.2%), heart failure (3.4% vs. 0.9%), atrial fibrillation (4.9% vs. 2%) and CKD (5.5% vs. 1.9%). HU showed a significant predictive value for CKD, stroke, CAD and myocardial Infarction. The overall mortality was also significantly higher in this group (8% vs. 2%). These findings support the hypothesis that HU may be a marker and predictor for cardiovascular morbidity and mortality. Having obtained these results, our findings are consistent with previous epidemiological studies, such as the Framingham Heart Study as a classical example, which described the associations between elevated uric acid levels and the occurrence of cardiovascular events as early as the 19th century [6] or more current meta-analyses, such as those by Zheng et al. and Zao et al., confirm these findings based on large cohorts [31,32]. Nakahashi et al. underline that progress in cardiovascular imaging having the ability to visualize monosodium urate crystals also in coronary vessels might enhance research and clinical decision making in coronary artery disease [33] so that maybe the role of HU in vascular calcification might become clearer and therapeutic impact might be driven more conclusively. In our cohort, Group 2 showed, in addition to elevated uric acid levels, a significantly higher prevalence of established risk factors, such as arterial hypertension (82.7% vs. 56.4%), obesity (mean BMI 29.8 vs. 26.1 kg/m^2^), diabetes mellitus (12.8% vs. 4.9%), dyslipidemia (48,1% vs. 54.2%), and impaired kidney function (eGFR 82 vs. 102 mL/min). These comorbidities are well-established components of the metabolic syndrome and are associated with the presence of HU [34,35]. Our observations reflect the pathophysiological vicious circle in which HU plays a significant role in the development and potentiation of metabolic syndrome [34,36,37]. HU exacerbates the progression of impaired kidney function, leading to the retention of uric acid and the accumulation of systemic inflammation and vascular damage, thereby closing the vicious cycle [38,39,40]. Evidence for this is the already reduced eGFR in Group 2 (82 mL/min vs. 102 mL/min) with a significantly higher incidence of CKD in Group 2 (5.6% vs. 1.6%). Although a direct causal relationship cannot be established in this observational cohort, our multivariable regression analysis indicates that HU remained independently associated with CKD after adjustment for major risk factors. This suggests that HU may contribute to renal impairment beyond traditional metabolic comorbidities, which is a new aspect and might serve as an additional explanation for the increased cardiovascular morbidity and mortality. Another aspect of the coexistence of HU and CKD includes the more complicated drug treatment if excretion of HU is impaired, especially due to reduced HU excretion from urine. In this cases, XOI are contraindicated in more severely impaired renal function but alternative agents such as febuxostat come to use and in very severe cases such as tumor lysis syndrome intravenous raspuricase could be applied.

Lifestyle factors such as meat or dietary habits and physical activity presented significant differences in our cohort. Group 2 showed a lower level of physical activity, which is a well-known risk factor for cardiovascular diseases [41]. Regular moderate to intensive physical activity improves insulin resistance and renal uric acid excretion [42,43,44]. Participants in Group 2 demonstrated a significantly different pattern of meat consumption compared to Group 1. This observation aligns with current evidence indicating that high meat intake, due to its elevated purine content, contributes to increased uric acid production and sustained elevation of serum uric acid levels [45,46]. Participants in Group 1 reported a significantly higher fruit consumption compared to those in Group 2. Although fructose-rich diet can acutely increase uric acid levels, the observed inverse association in our cohort appears to be a marker for balanced nutrition and healthier lifestyle [47,48].

The relationship between HU and cognitive function remains controversial: while some studies suggest neuroprotective effects, others link HU to increased dementia risk due to vascular damage. In our cohort, cognitive performance improved in both groups [49,50,51,52]. These changes are more likely attributable to an overall improvement of cardiovascular risk profile rather than a direct effect of serum uric acid [53].

The strengths of the study include the large sample size, detailed collection of laboratory and lifestyle data, and the prospective study design.

Potential limitations include the interventionist approach as it is possible that endpoint biases were introduced due to improvements in cardiovascular risk profiles of both groups, but especially Group 2. Second, as an observational study, causal relationships cannot be inferred. Third, dietary and physical activity data were self-reported and therefore subject to recall bias. Over- or underestimation of these behaviors cannot be ruled out. Additional baseline imbalances between the two groups may have biased follow-up risk estimates. For example, participants with HU had higher prevalence prior stroke, which increases risk for subsequent events.

In summary, our results show a significant association between HU and cardiovascular complications. The observed relationships cannot be interpreted independently of accompanying risk factors, meaning that a definitive causal relationship cannot be established. Given the observed clustering of metabolic dysfunctions, including glucose intolerance, obesity, hypertension, and dyslipidemia, in combination with existing HU, the routine measurement of elevated uric acid levels in clinical practice could represent a valuable tool for the early identification of high-risk cardiovascular patients. Particularly in patients with multiple metabolic risk factors, HU should be considered as a potential exacerbator of overall risk. Whether serum uric acid reduction through early use of XOIs leads to a reduction in cardiovascular events remains unclear and requires further studies. A renewed prospective interventional study over an extended period would be advisable. While current guidelines recommend therapy only for symptomatic HU or organ manifestation, our results support a more individualized approach to cardiovascular risk profiles, particularly in high-risk populations, with potential early use of XOIs even before organ manifestation.

## Figures and Tables

**Table 1:** Baseline characteristics.

	Group 1	Group 2	*p*-Value
Participants *n* (%)	3288 (80.3)	805 (19.7)	<0.001
Age mean (SD) in years	51.8 (13.6)	58.0 (14.1)	<0.001
Male, *n* (%)	1589 (48.5)	490 (60.9)	<0.001
Female, *n* (%)	1688 (51.5)	315 (39.1)	<0.001
Serum uric acid, mean (SD) in mg/dL	4.8 (1.03)	7.1 (1.13)	<0.001
Serum uric acid level > 6.5 mg/dL, *n* (%)		743 (92.2)	<0.001
Serum uric acid > 10 mg/dL, *n* (%)		13 (1.6)	<0.001
Diabetes mellitus, *n* (%)	146 (4.9)	103 (12.8)	<0.001
Arterial Hypertension, *n* (%)	1847 (56.4)	666 (82.7)	<0.001
BMI > 30 kg/m^2^ *n* (%)	553 (16.9)	350 (42.5)	<0.001
Tobacco use, *n* (%)	467 (14.3)	91 (11.3)	0.116
HbA1C * %, mean (SD) in %	5.2 (0.56)	5.6 (0.65)	<0.001
LDL *, mean (SD) in mg/dL	128.9 (35.4)	136.9 (34.9)	<0.001
HDL *, mean (SD) in mg/dL	63 (18.7)	52.6 (15.6)	<0.001
Lp(a) *, mean (SD) in nmol/L	42.1 (20.3)	43.4 (16.4)	<0.001
eGFR *, mean (SD) in mL/min	102.7 (24.5)	82.1 (26.4)	<0.001
Stroke, *n* (%)	54 (1.64)	28 (3.4)	0.002
CAD *, *n* (%)	96 (2.9)	75 (9.3)	<0.001
MI *, *n* (%)	38 (1.15)	40 (4.9)	<0.001
HF *, *n* (%)	56 (1.7)	36 (4.4)	<0.001
AF *, *n* (%)	94 (2.8)	55 (6.8)	<0.001
PAD *, *n* (%)	154 (4.7)	59 (7.3)	0.003
CKD *, *n* (%)	63 (1.92)	114 (14.1)	<0.001
Regular physical activity, *n* (%)	1630 (43.1)	275 (33.5)	<0.001
Moderate physical activity, *n* (%)	1081 (28.6)	244 (29.6)	<0.001
Sparse physical activity, *n* (%)	1071 (28.3)	301 (36.7)	<0.001
High fruit intake, *n* (%)	2221 (58.7)	460 (56.1)	<0.001
Moderate fruit intake, *n* (%)	934 (24.7)	205 (25.0)	0.104
Low fruit intake, *n* (%)	579 (15.3)	142 (17.3)	0.27
High meat intake, *n* (%)	900 (13.3)	186 (22.7)	0.016
Moderate meat intake, *n* (%)	2159 (65.0)	494 (61.3)	0.024
Low meat intake, *n* (%)	686 (20.9)	129 (16.0)	0.002
DemTect baseline, mean (SD) in points	16.3 (2.2)	15.5 (2.8)	
DemTect follow-up, mean (SD) in points	16.6 (2.2)	16.4 (2.2)	

* CAD; coronary artery disease, CKD; chronic kidney disease, PAD; peripheral artery disease, AF; atrial fibrillation, HF; heart failure, MI; myocardial infarction, BMI; body mass index, HbA1C; glycated hemoglobin, LDL; low-density lipoprotein cholesterol, HDL; high-density lipoprotein, Lp(a); lipoprotein(a), eGFR; estimated glomerular filtration rate.

**Table 2:** Endpoints.

	Group 1	Group 2	*p*-Value
CAD *, *n* (%)	108 (3.3)	67 (8.4)	<0.001
HF *, *n* (%)	31 (0.9)	27 (3.4)	<0.001
Stroke *, *n* (%)	40 (1.2)	21 (2.6)	<0.001
MI *, *n* (%)	44 (1.3)	25 (3.1)	<0.001
PAD *, *n* (%)	77 (2.4)	36 (4.5)	<0.001
AF *, *n* (%)	67 (2.0)	39 (4.9)	<0.001
CKD *, *n* (%)	62 (1.9)	44 (5.5)	<0.001

* CAD; coronary artery disease, CKD; chronic kidney disease, PAD; peripheral artery disease, AF; atrial fibrillation, HF; heart failure, MI; myocardial infarction.

**Table 3:** Multivariate logistic regression analysis: significance levels (*p*-values) of age, diabetes mellitus, and hyperuricemia as predictors.

	Age	Diabetes Mellitus	Hyperuricemia
CAD *, *p*-Value	<0.001	<0.001	<0.001
HF *, *p*-Value	<0.001	0.018	0.092
Stroke *, *p*-Value	<0.001	<0.001	0.049
PAD *, *p*-Value	<0.001	0.526	0.115
AF *, *p*-Value	<0.001	0.414	0.441
CKD *, *p*-Value	<0.001	0.015	0.022
MI *, *p*-Value	<0.001	0.011	0.003

* CAD; coronary artery disease, CKD; chronic kidney disease, PAD; peripheral artery disease, AF; atrial fibrillation, HF; heart failure, MI; myocardial infarction.

## Data Availability

Additional and raw data can be obtained from the corresponding author under fady.al-azem@uni-oldenburg.de.

## Abbreviations

The following abbreviations are used in this manuscript:

AF	Atrial Fibrillation
BMI	Body mass index
CAD	Coronary artery disease
CKD	Chronic kidney disease
CVRF	Cardiovascular risk factors
eGFR	Estimated glomerular filtration rate
HbA1c	Glycated hemoglobin
HDL	High-density lipoprotein
HF	Heart failure
HU	Hyperuricemia
KDIGO	Kidney Disease: Improving Global Outcomes
LDL	Low-density lipoprotein
Lp(a)	Lipoprotein (a)
MI	Myocardial Infarction
PAD	Peripheral artery disease
SD	Standard deviation
SPSS	Statistical Package for the Social Sciences
XOI	Xanthine oxidase inhibitor
